# Supplementary material for: Venovenous bypass in adult liver transplant recipients: A single-center observational case series
Source: PLoS One. 2024 May 31;19(5):e0303631. doi: 10.1371/journal.pone.0303631 (PMC11142538; doi:10.1371/journal.pone.0303631)
Supplement: S1 File — Data are presented as median (interquartile range) and [minimum maximum]. (DOCX) [file pone.0303631.s001.docx]

**Supplementary File 1. Table 1.** Intraoperative arterial blood gas data for veno-venous bypass patients. Data are presented as median (interquartile range) and [minimum maximum].

|  | **Phase I** | **Phase II** | **Phase III** |
| --- | --- | --- | --- |
| **FiO_2_** | 0.50 (0.21 - 0.6) [0.21 - 1] | 0.50 (0.45 - 0.6) [0.21 - 1] | 0.50 (0.40 - 0.53) [0.21 - 0.70] |
| **pH** | 7.36 (7.33 - 7.41) [7.24 - 7.53] | 7.37 (7.33 - 7.39) [7.23 - 7.56] | 7.30 (7.25 - 7.35) [7.12 - 7.55] |
| **pO_2_ (mmHg)** | 249.5 (192.8 - 298.5) [111.0 - 538.0] | 262 (206.3 - 332) [136 - 539] | 242 (177.8 - 294.8) [80.8 - 527] |
| **pCO_2_ (mmHg)** | 38.2 (33.3 - 41.0) [25 - 51] | 35.3 (32.8 - 37) [28 - 43] | 40.5 (35 - 43) [32 - 49] |
| **HCO_3_^-^ (mmol/L)** | 22 (19.2 - 24.5) [16 - 27.8] | 19.6 (17.8 - 21.1) [13 - 24] | 18.9 (16.3 - 22.0) [13 - 29] |
| **Oxygen saturation (%)** | 100 (99.8 - 100) [29 - 100] | 100 (99.8 - 100) [97 - 100] | 100 (99 - 100) [97 - 100] |
| **Sodium (mmol/L)** | 138 (135 - 140) [132 - 152] | 137.5 (133.8 - 140) [129 - 148] | 138 (136 - 141.5) [132 - 146] |
| **Potassium (mmol/L)** | 4.2 (3.8 - 4.4) [3.3 - 5] | 4.2 (3.9 - 4.5) [3.3 - 5] | 3.8 (3.6 - 4.3) [3.0 - 6.5] |
| **Chloride (mmol/L)** | 106 (100.5 - 109) [96 - 120] | 104 (101 - 108) [95 - 119] | 102 (99.5 - 107) [97 - 117] |
| **Calcium (mmol/L)** | 1.08 (0.99 - 1.13) [0.84 - 1.31] | 1.02 (0.90 - 1.15) [0.70 - 1.26] | 1.19 (1.06 - 1.36) [0.58 - 1.59] |
| **Hemoglobin (g/L)** | 80.5 (69.5 - 94.5) [57 - 130] | 81.5 (68.5 - 92) [57 - 127] | 86 (75.5 - 95.8) [53 - 122] |
| **Glucose (mmol/L)** | 6.1 (4.8 - 7) [4.1 - 8.2] | 5.5 (4.4 - 6.1) [1.8 - 8.8] | 9.3 (7 - 10.2) [2.3 - 12.4] |
| **Lactate (mmol/L)** | 1.35 (0.78 - 4.38) [0.4 - 11] | 3 (2.4 - 7.8) [1.8 - 13.8] | 5.1 (3.4 - 8.4) [1.1 - 12.7] |

**Supplementary 1 File Table 2.** Postoperative arterial blood gas data for veno-venous bypass patients. Data are presented as median (interquartile range) and [minimum maximum].

|  | **On Arrival** | **At 24hrs** | **At 48hrs** |
| --- | --- | --- | --- |
| **FiO_2_** | 0.50 (0.30 - 1.0) [0.21 - 1.0] | 0.21 (0.21 - 0.30) [0.21 - 0.50] | 0.21 (0.21 - 0.30) [0.21 - 0.50] |
| **pH** | 7.37 (7.29 - 7.42) [7.22 - 7.49] | 7.42 (7.38 - 7.45) [7.34 - 7.50] | 7.43 (7.41 - 7.45) [7.32 - 7.48] |
| **pO_2_ (mmHg)** | 267 (127.5 - 455.3) [79 - 577] | 101.5 (77.8 - 115.8) [41.0 - 159.0] | 96.5 (80.3 - 111) [30 - 191] |
| **pCO_2_ (mmHg)** | 40.0 (36.3 - 42.8) [28 - 55] | 38.5 (37.0 - 41.8) [32.0 - 50.0] | 39.5 (35.3 - 42.8) [30 - 50] |
| **HCO_3_^-^ (mmol/L)** | 22.1 (20.1 - 24.8) [14 - 28] | 25.2 (23 - 27.8) [19.0 - 31.0] | 26 (22.3 - 27.1) [19 - 31] |
| **Oxygen saturation (%)** | 100 (99 - 100) [96 - 100] | 98 (96 - 99) [28 - 100] | 97.5 (96 - 99) [55 - 100] |
| **Sodium (mmol/L)** | 137 (136.3 - 139) [131 - 143] | 127 (134 - 139.8) [128 - 145] | 135.5 (131.5 - 138) [127 - 145] |
| **Potassium (mmol/L)** | 4.1 (3.9 - 4.4) [3.7 - 5.9] | 4.1 (3.7 - 4.3) [3.1 - 4.8] | 4.0 (3.8 - 4.2) [3.4 - 4.7] |
| **Chloride (mmol/L)** | 102 (101 - 106.8) [98 - 113] | 103 (101.3 - 107) [97 - 111] | 103.5 (101.3 - 106) [94 - 113] |
| **Calcium (mmol/L)** | 1.16 (1.04 - 1.22) [0.94 - 1.32] | 1.14 (1.10 - 1.25) [1.02 - 1.46] | 1.14 (1.09 - 1.23) [1.04 - 1.42] |
| **Hemoglobin (g/L)** | 96 (82.8 - 116.8) [76 - 135] | 81 (74.8 - 91) [65 - 121] | 84.5 (78.3 - 92.8) [71 - 110] |
| **Glucose (mmol/L)** | 10.1 (8.4 - 12.5) [4.7 - 19.3] | 8.7(7.3 - 10.2) [5.3 - 12.8] | 8.2 (6.8 - 10.8) [5.4 - 13.5] |
| **Lactate (mmol/L)** | 2.9 (1.1 - 6.3) [0.8 - 14.8] | 1.5 (1.0 - 2.9) [0.7 - 9.7] | 1.4 (1.0 - 2.3) [0.6 - 4.4] |
